# Application of Quantitative MRI for Brain Tissue Segmentation at 1.5 T and 3.0 T Field Strengths

**DOI:** 10.1371/journal.pone.0074795

**Published:** 2013-09-16

**Authors:** Janne West, Ida Blystad, Maria Engström, Jan B. M. Warntjes, Peter Lundberg

**Affiliations:** 1 Center for Medical Image Science and Visualization, Linköping University, Linköping, Sweden; 2 Radiation Physics, Department of Medicine and Health, Linköping University, UHL County Council of Östergötland, Linköping, Sweden; 3 Radiology, Department of Medicine and Health, Linköping University, UHL County Council of Östergötland, Linköping, Sweden; 4 Division of Clinical Physiology, Department of Medicine and Health, Linköping University, Linköping, Sweden; Tokyo Metropolitan Institute of Medical Science, Japan

## Abstract

**Background:**

Brain tissue segmentation of white matter (WM), grey matter (GM), and cerebrospinal fluid (CSF) are important in neuroradiological applications. Quantitative Mri (qMRI) allows segmentation based on physical tissue properties, and the dependencies on MR scanner settings are removed. Brain tissue groups into clusters in the three dimensional space formed by the qMRI parameters R_1_, R_2_ and PD, and partial volume voxels are intermediate in this space. The qMRI parameters, however, depend on the main magnetic field strength. Therefore, longitudinal studies can be seriously limited by system upgrades. The aim of this work was to apply one recently described brain tissue segmentation method, based on qMRI, at both 1.5 T and 3.0 T field strengths, and to investigate similarities and differences.

**Methods:**

In vivo qMRI measurements were performed on 10 healthy subjects using both 1.5 T and 3.0 T MR scanners. The brain tissue segmentation method was applied for both 1.5 T and 3.0 T and volumes of WM, GM, CSF and brain parenchymal fraction (BPF) were calculated on both field strengths. Repeatability was calculated for each scanner and a General Linear Model was used to examine the effect of field strength. Voxel-wise t-tests were also performed to evaluate regional differences.

**Results:**

Statistically significant differences were found between 1.5 T and 3.0 T for WM, GM, CSF and BPF (p<0.001). Analyses of main effects showed that WM was underestimated, while GM and CSF were overestimated on 1.5 T compared to 3.0 T. The mean differences between 1.5 T and 3.0 T were -66 mL WM, 40 mL GM, 29 mL CSF and -1.99% BPF. Voxel-wise t-tests revealed regional differences of WM and GM in deep brain structures, cerebellum and brain stem.

**Conclusions:**

Most of the brain was identically classified at the two field strengths, although some regional differences were observed.

## Introduction

Brain tissue segmentation and volume estimation of white matter (WM), grey matter (GM), and cerebrospinal fluid (CSF) are important in many neuroradiological applications [1,2,3]. Volume estimation of segmented brain tissue can be used to assess local and global brain atrophy that is present in diseases such as multiple sclerosis [1,4], Alzheimer’s disease [5] and other forms of dementia [6], and this measure can in turn be used to monitor neurodegenerative disease progression [5,7], as well as to monitor the effects of therapy and rehabilitation [8].

There are two general approaches to automated brain tissue segmentation; either (1) each voxel is assigned to one specific tissue class [9,10,11,12], or (2) each voxel is assigned volume fractions of several different tissue classes [13,14,15]. Allowing more than one tissue class in each voxel has advantages since partial volume is common in the brain, even at high-resolution imaging, and especially close to tissue borders, such as in the cortex where voxels often contain mixtures of GM and WM, or CSF. Allowing only one tissue class in each voxel is prone to partial-volume errors. Furthermore, calculating tissue fractions decreases the dependence on high-resolution imaging, something that requires a large signal-to-noise-ratio (SNR).

Most brain tissue segmentation methods are based on conventional contrast-weighted MR images [10,11,12,13,14,15]. Although these images provide high neuro-anatomical detail, segmentation is complicated by contrast inhomogeneities and the arbitrary grey-scale of the images. Furthermore a variety of experimental parameters influence conventional contrast-weighted MR images, such as the repetition time (TR), echo time (TE), and inversion delay time (TI). The complexity of the acquired images problematises physical interpretation of the signal intensity. Therefore, segmentation is often performed using contrast differences in the image, and not the absolute pixel values. Different normalisation strategies or *ad hoc* filters may be necessary to compensate for variations of signal intensity in the imaged volume (see 10 or [15]).

Conversely, quantitative MRI (qMRI) allows the measurements of physical tissue parameters such as the relaxation rates R_1_ (inverse of the relaxation time T_1_, 1/T_1_) and R_2_ (1/T_2_), and the proton density (PD) [16,17,18,19,20,21,22,23]. By using qMRI for tissue segmentation, the dependence on MR scanner hardware and settings is largely removed. This allows for segmentation based on physical tissue characteristics [24]. In recent years quantification methods that are feasible within clinically acceptable scanning times have been presented [18,22]. It has also been shown that human brain tissue can be differentiated using qMRI parameters [9,25] and that tissues group into clusters in the three-dimensional space formed by R_1_, R_2_ and PD (the R_1_-R_2_-PD space) [22,24]. In those voxels where two tissues are present, both tissue types contribute to the MR-signal, and quantification results in a weighted average of the tissues’ individual R_1_, R_2_ and PD values. The qMRI parameters describing the relaxation rates of tissue, however, depend on the main magnetic field strength. The R_1_ and R_2_ parameters generally decrease when the main magnetic field strength is increased; this effect is not linear and depends on tissue composition. In particular, signal alteration in tissues rich in iron increases due to the ferromagnetic properties of the iron, causing decreased R_1_ and R_2_ [26,27,28].

Because of this, longitudinal studies, as well as quantitative patient follow-ups, can be seriously limited by system changes from lower to higher field strengths. Many widely used segmentation tools, such as FreeSurfer [29] and Bayesian based methods [30], report variable results across field strengths in combination with conventional MRI [27,31], and combining data acquired at different field strengths has for this reason not been endorsed. Studies have reported regional differences across field strengths when using automatic segmentation methods [27,31,32], as well as overall volume differences of brain tissues and intracranial volume [33]. One study by Jovicich et al reported differences of up to 10% for total intracranial volume when using the FreeFurfer tool to compare 1.5 T and 3.0 T [31]. In another study Keihaninejad et al found differences of up to 12.5% using Bayesian methods and up to 14.7% using SPM, also comparing intracranial volume between 1.5 T and 3.0 T systems. Moreover, one study even reported systematic volume differences when manual segmentation was used [33].

The aim of this work was to apply one recently described brain tissue segmentation method based on qMRI [24] at both 1.5 T and 3.0 T field strengths, and to investigate the differences and potential value of combining field strengths in research studies and clinical practice.

## Materials and Methods

### Overview

**Figure 1:**
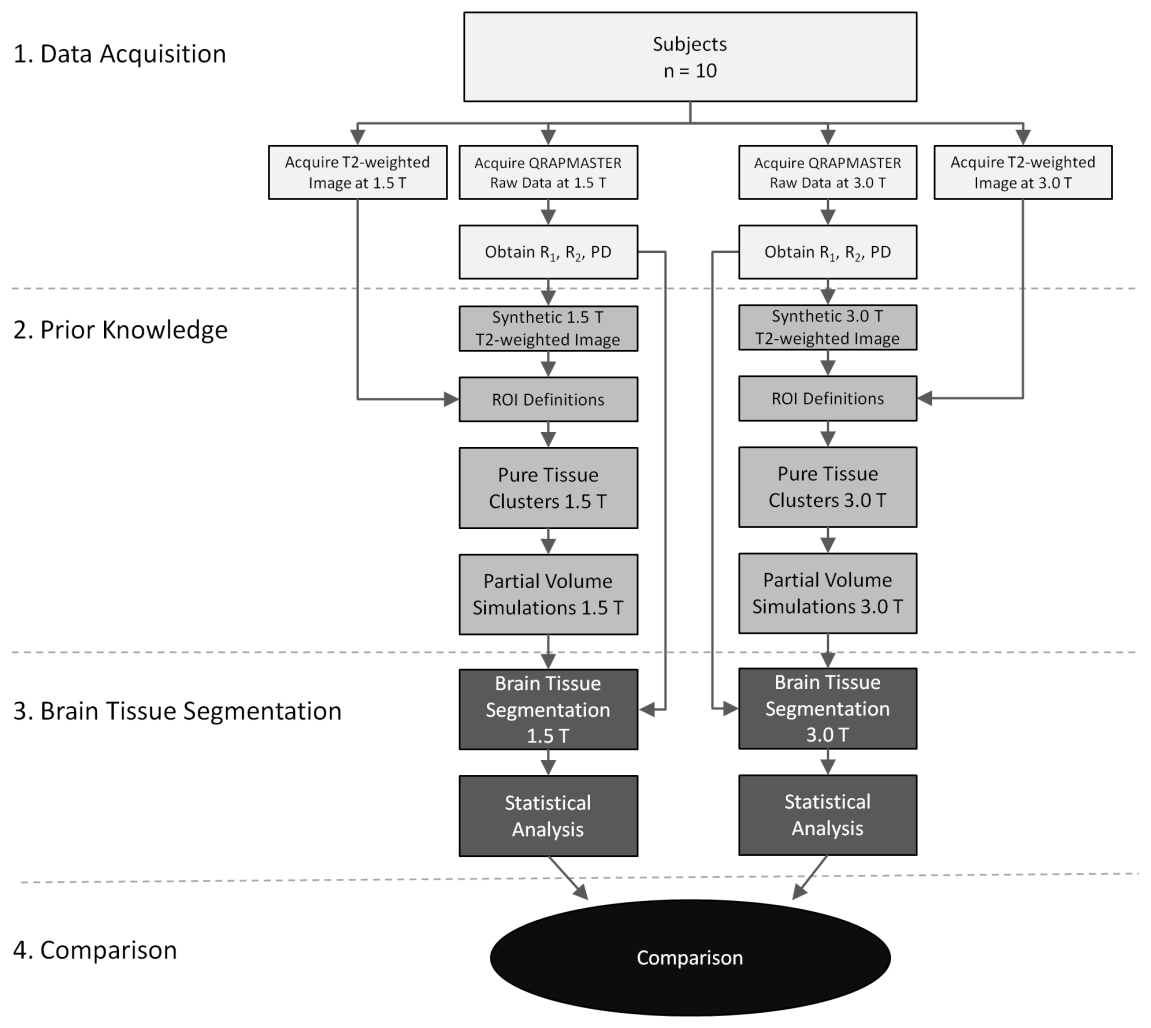
Flow chart shows the different steps performed in this study. First **(1)** data acquisition was performed on 10 healthy subjects at both 1.5 T and 3.0 T field strengths **(2)**, prior knowledge required in the segmentation procedure were determined **(3)**, the brain tissue segmentation procedure were performed, and finally **(4)** the results were compared between the field strengths.

In this work one brain tissue segmentation method, previously only reported for 1.5 T, was implemented for both 1.5 T and 3.0 T scanner systems [24]. Data acquisition was performed on 10 healthy subjects at both field strength, and results were compared. The segmentation method required prior knowledge about (1) ‘pure tissue clusters’ in the three dimensional space formed by using R_1_, R_2_ and PD as basis (the R_1_-R_2_-PD space) and (2) the characteristics of partial volume voxels in this space. Prior to performing the brain tissue segmentation data from the 10 healthy subjects were used to obtain this prior knowledge, which was then used to calibrate the segmentation method. Pure tissue clusters were determined by region of interest (ROI) measurements, and the characteristics of partial volume voxels were estimated with simulations. In the simulations the measured pure tissue cluster values were combined to create synthetic tissue mixtures. Finally; data from the 10 healthy subjects were segmented and results from the two field strengths were compared. The study design is illustrated in Figure 1.

### Data Acquisition

In vivo measurements were performed with a 1.5 T Philips Achieva scanner (software version 3.2.1), using an 8-channel SENSE head coil, and a 3.0 T Philips Ingenia scanner (software version 4.1.1), using an 16-channel SENSE head coil, on 10 healthy subjects (four males, six females, aged 24.4 ± 2.5, range 21 to 29 years). Health status was assessed using a questionnaire where brain disorders were ruled out. QMRI data were acquired using the multi-slice, multi-echo and multi-saturation delay sequence ‘QRAPMASTER’ (also known as qMAP) [22]. Whole-brain qMRI maps of the tissue parameters R_1_, R_2_, and PD were calculated using SyMRI Diagnostics (0.9.11) software (SyntheticMR AB, Sweden, 2012) from the acquired raw data. The qMRI sequence settings were; repetition time (TR) = 5000 ms, saturation delay times (TD) = 132/632/2257/4882 ms, echo times (TE) = 16/32/48/64/80/96 ms. Voxel size was 3x1.3x1.3 mm^3^ and a gap of 0.5 mm was applied in order to reach whole-brain coverage. The sequence was identical for 1.5 T and 3.0 T, and each scan took 6 minutes. For additional details on the QRAPMASTER qMRI sequence see 22. In addition, conventional T2-weighted images were acquired. For each of the two scanners, the subjects were scanned in two separate sessions, and were removed from the scanner room between these sessions. In each session, two acquisitions were performed. This resulted in a total of eight data sets for each subject (two scanners * two sessions * two acquisitions). The study was approved by the Regional Ethical Review Board in Linköping (Dnr: M88-07 T93-08) and written informed consent was obtained from all subjects prior to study entry.

### Prior Knowledge - Pure Brain Tissue Clusters

In order to measure the pure brain tissue clusters in R_1_-R_2_-PD space, required as prior knowledge in the segmentation procedure, a neuroradiologist (IB) manually positioned standardised regions of interest (ROIs). The ROI size was 3x3 voxels (corresponding to 3.9x3.9 mm^2^) and the ROIs were placed on synthetic T2-weighted images with conventional T2-weighted images used as a visual reference. Synthetic T2-weighted images were generated from the quantitative R_1_-R_2_-PD data using the SyMRI Diagnostics (0.9.11) software (SyntheticMR AB, Sweden, 2012) as described elsewhere [22]. Synthetic T2-weighted images were generated from the same data as the qMRI parameter maps and therefore perfectly registered, removing the need for additional data registration. Both 1.5 T and 3.0 T image ROIs were placed in the same session in order to assure equivalent ROI placement on images from both field strengths. ROIs were placed on both datasets from the first session of each subject in each scanner, resulting in a total of four datasets for each individual subject (two for each of the field strengths). ROIs were placed in the structures of GM, WM and CSF. GM R_1_-R_2_-PD was measured para-sagittally in the frontal lobe, anterior to the genu of the corpus callosum where the cortex is thick (left and right); CSF was measured in the frontal horns (left and right); WM was measured peri-triagonally (left and right), frontally (left and right), parietally in the centrum semiovale (left and right), and in the genu and splenium of the corpus callosum. The average ± SD for R_1_, R_2_ and PD of the tissue types was calculated from all the voxels in the ROIs, for each tissue type. For WM, each tissue region was calculated separately, as was an average of all eight WM ROIs.

### Prior Knowledge - Partial Volume Simulations

In order to estimate the characteristics of partial volume voxels in R_1_-R_2_-PD space, required as prior knowledge in the segmentation procedure, a numerical Bloch simulator that could simulate mixtures of different tissue types was used. Mathematical details are described in Appendix S1.

The Bloch simulator was used to estimate the observed R_1_, R_2_ and PD values for combinations of two tissues in the transitions WM↔GM, WM↔CSF and GM↔CSF, for both 1.5 T and 3.0 T field strengths separately. When two tissues were combined, the tissue content was altered in incremental steps of 0.1% to cover a range of 0 to 100% of each tissue type. The Bloch simulation with the QRAPMASTER sequence was executed three times for each of the two field strengths, with R_1_ and R_2_ values from the three previously measured pure brain tissue clusters, in order to acquire the magnetization states, E_*tissue*_, for WM, GM and CSF. For each specific partial volume mixture of two tissues, the magnetisation state matrices, E_*tissue1*_ and E_*tissue2*_, were combined and weighted with the PD and the volume fraction, α, of the tissues according to:

$$E_{combined}=\alpha *PD_{tissue1}*E_{tissue1}+(1-\alpha )*PD_{tissue2}*E_{tissue2}$$ (1)

This combination of two magnetization states yields the sum of the two tissues assuming that each tissue generates signals independent of the other and that there is no interaction between the tissues in this approximation.

In order to retrieve R_1_, R_2_ and PD for the mixture the QRAPMASTER fitting in the software SyMRI Diagnostics was used, for additional details on the fitting process see 22.

The partial volume simulations were all included in one Monte Carlo simulation and repeated 10,000 times, with the SNR set to 20 (corresponding to the noise setting suggested in Ref. [24]).

As tissue fractions were altered in 0.1% steps in the three partial volume transitions, the Monte Carlo simulation resulted in 3,000 multivariate tissue clusters in the R_1_–R_2_–PD space, for both field strengths separately. These clusters were tested for normal distribution, using skewness and kurtosis tests. The skewness is a measure of the asymmetry of a distribution, and the kurtosis is a measure of the relative concentration of data values, in the centre versus in the tails, of a distribution. For normal distribution the skewness and kurtosis are zero, but any value below 2 suggests that the data are normally distributed [34]. For normal distribution data, covariance matrices were calculated. These simulated tissue mixtures were used to describe partial volume characteristics in the segmentation procedure.

### Brain Tissue Segmentation

Once the pure tissue clusters and the relation between partial volume tissue composition and corresponding R_1_, R_2_, PD values were established, this prior knowledge was used to construct tissue ‘lookup grids’ in the R_1_-R_2_-PD space, for 1.5 T and 3.0 T. The lookup grids translated in vivo qMRI data into tissue volume fractions of WM, GM and CSF using the R_1_, R_2_ and PD values by comparison to the prior knowledge. The lookup grids were constructed such that in vivo voxels with R_1_, R_2_ and PD values within the 95% prediction ellipses of the measured pure tissue clusters for WM, GM and CSF, were mapped to 100% pure tissue. All the remaining simulated clusters, with different partial volume fractions of two tissues, were overlaid on the lookup grid. Since the change in voxel mixture in the simulations were performed using 0.1% incremental steps, these clusters appeared close together and overlapped. In vivo voxels with R_1_, R_2_ and PD values within the 95% prediction ellipses of the simulated partial-volume tissue clusters were mapped to the tissue fractions of the closest simulated cluster. Remaining in vivo voxels, outside the defined lookup grids in R_1_-R_2_-PD space, were considered undefined and termed ‘Non-WM/GM/CSF’ (or ‘NON’). Brain segmentation was implemented in C++ as a module to SyMRI Diagnostics.

### Statistical Analysis

For all acquisitions at 1.5 T and 3.0 T, volumes of WM, GM, CSF and NON were calculated using the corresponding lookup grid. The ‘Brain Parenchymal Volume’ (BPV) was calculated as the sum of WM, GM and NON. The ‘Intracranial Volume’ (ICV) was calculated as the sum of BPV and CSF, and finally the ‘Brain Parenchymal Fraction’ (BPF) was calculated as the ratio of BPV to ICV. Volume measurements were tested for normal distribution using the Shapiro-Wilk’s test. For images acquired in the first acquisition of each session the within-subject standard deviation, *sw*, was estimated as the square root of the mean within-subject variance. The repeatability was calculated as 2.77 * *sw*, as suggested by Bland and Altman, see 35. This definition of ‘repeatability’ was based on the assumption that the difference between any two measurements of the same subject is expected to be less than this for 95% of pairs of observations. Finally, the within-subject coefficient of variation (CV) was calculated.

The General Linear Model (GLM) was used to examine the effect of field strength on the determined volume measures. Field strength, subject and session were included as fixed factors in the model. Analyses of main effects were performed where significance was found. Statistical analyses were performed using SPSS Statistics 19 (IBM, USA, 2010).

In order to investigate regional differences in tissue characteristics between the 1.5 T and 3.0 T scanners, voxel-wise t-tests of each tissue type were performed using SPM8 (Wellcome Department of Imaging Neuroscience, University College, London, UK). Before calculating the statistics, maps of WM, GM, and CSF were normalized to a standard stereotactic space in Montreal Neurological Institute (MNI) coordinates using the T2-weighted template available in SPM8. Synthetic T2-weighted images, which were smoothed with an 8 mm Gaussian kernel to reduce the individual anatomical details, were used as source images to calculate the transformation matrix. A 12-parameter (translation, rotation, shear, zoom) affine registration followed by nonlinear deformations, defined by a linear combination of three dimensional discrete cosine basis functions, were used to transform the synthetic T2-weighted images of each subject to the template. The resulting transformation matrices were then applied to the WM, GM, and CSF maps. The resulting maps were re-gridded to 2 x 2 x 2 mm^3^ voxel size to obtain an isotropic dataset. Voxel-wise differences between maps acquired at 1.5 T and 3.0 T were estimated by two-tailed paired t-tests of each tissue type. The resulting t-maps were initially thresholded at p=0.001, uncorrected for multiple comparisons. Based on those initially thresholded maps, ROI analyses were made of regions representing the different divisions of the brain: the frontal, temporal, parietal, occipital, and the limbic lobes; the latter including the cingulate cortex and the hippocampal formation; as well as the cerebellum (anterior and posterior lobes), pons, medulla, midbrain, i.e. the upper brain stem, and the sub-lobar regions comprising the thalamus and the basal ganglia. The results are reported as significant if cluster p<0.001, Family Wise Error (FWE) corrected, and cluster size was at least 20 voxels.

## Results

### Prior Knowledge

The presented brain tissue segmentation method required prior knowledge on pure brain tissue cluster positions, which were measured using ROIs. Results from these measurements are presented in Table 1 for both 1.5 T and 3.0 T. These values were used in the subsequent partial volume simulations and in order to create the lookup grids, which subsequently were used to segment the *in vivo* data.

**Table 1:** qMRI tissue parameters from manually placed regions of interest in 10 healthy subjects, on both 1.5 T and 3.0 T systems. n indicates the number of ROI’s used per region in each subject.

		**1.5 T**			**3.0 T**		
**Anatomy**	**n**	**R_1_ (s^-1^**)	**R_2_ (s^-1^**)	**PD (%**)	**R_1_ (s^-1^**)	**R_2_ (s^-1^**)	**PD (%**)
Frontal white matter	2	1.67 ± 0.09	13.55 ± 0.63	64.86 ± 2.04	1.39 ± 0.06	14.49 ± 0.57	66.31 ± 1.46
Peritrigonal white matter	2	1.64 ± 0.12	12.66 ± 0.90	62.03 ± 3.07	1.41 ± 0.08	13.57 ± 0.89	63.14 ± 2.90
Centrum Semiovale	2	1.51 ± 0.08	11.80 ± 0.56	68.53 ± 1.75	1.31 ± 0.04	12.77 ± 0.56	68.27 ± 1.51
Genu	1	1.78 ± 0.14	14.23 ± 0.73	59.88 ± 2.26	1.46 ± 0.07	15.29 ± 0.58	63.72 ± 1.46
Splenium	1	1.58 ± 0.12	12.46 ± 0.79	65.33 ± 2.92	1.38 ± 0.09	13.11 ± 0.83	65.61 ± 2.37
Average white matter	8	1.62 ± 0.14	12.84 ± 1.09	64.51 ± 3.77	1.38 ± 0.08	13.76 ± 1.10	65.60 ± 2.84
Frontal Cortex	2	0.81 ± 0.08	10.29 ± 0.59	88.06 ± 3.76	0.77 ± 0.08	10.69 ± 0.76	86.88 ± 2.86
CSF	2	0.24 ± 0.01	0.87 ± 0.55	103.76 ± 3.54	0.32 ± 0.09	0.79 ± 0.59	94.40 ± 6.59

**Figure 2:**
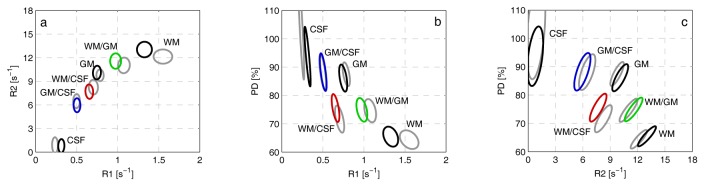
Pure tissue clusters WM, GM, CSF, and the mixtures of 50% WM/GM, 50% WM/CSF and 50% GM/CSF shown in the R1-R2-PD space. Projections on ***a*** **)** R1-R2, ***b*** **)** R1-PD and ***c*** **)** R2-PD planes. The contour lines show the 95% prediction ellipses for simulations with 20. Coloured ellipses are for 3.0 T and corresponding grey clusters are for 1.5 T. This figure shows the differences in tissue characteristics between 1.5 T and 3.0 T field strengths.

The partial volume simulations resulted in 3,000 R_1_-R_2_–PD clusters for each field strength, one for each specific mixture of two tissue types in the transitions WM↔GM, WM↔CSF and GM↔CSF. Results from the simulations at both field strengths are shown in Figure 2. In this figure only the pure tissue clusters and 50% mixtures are presented. These clusters are shown as projections on the R_1_-R_2_, R_1_-PD and R_2_-PD planes, the indicated ellipses enclose 95% of the R_1_–R_2_–PD estimates from the Monte Carlo simulation. The observed clusters are not tilted in the R_1_–R_2_ plot, suggesting that the correlation between R_1_ and R_2_ is low. In the R_1_–PD and the R_2_–PD plots; however, the clusters are tilted, which indicates a higher correlation of R_1_ and R_2_ with PD. Skewness and kurtosis measurements indicated that the tissue clusters were normal distributed in the R_1_-R_2_-PD space.

### Brain Tissue Segmentation

**Figure 3:**
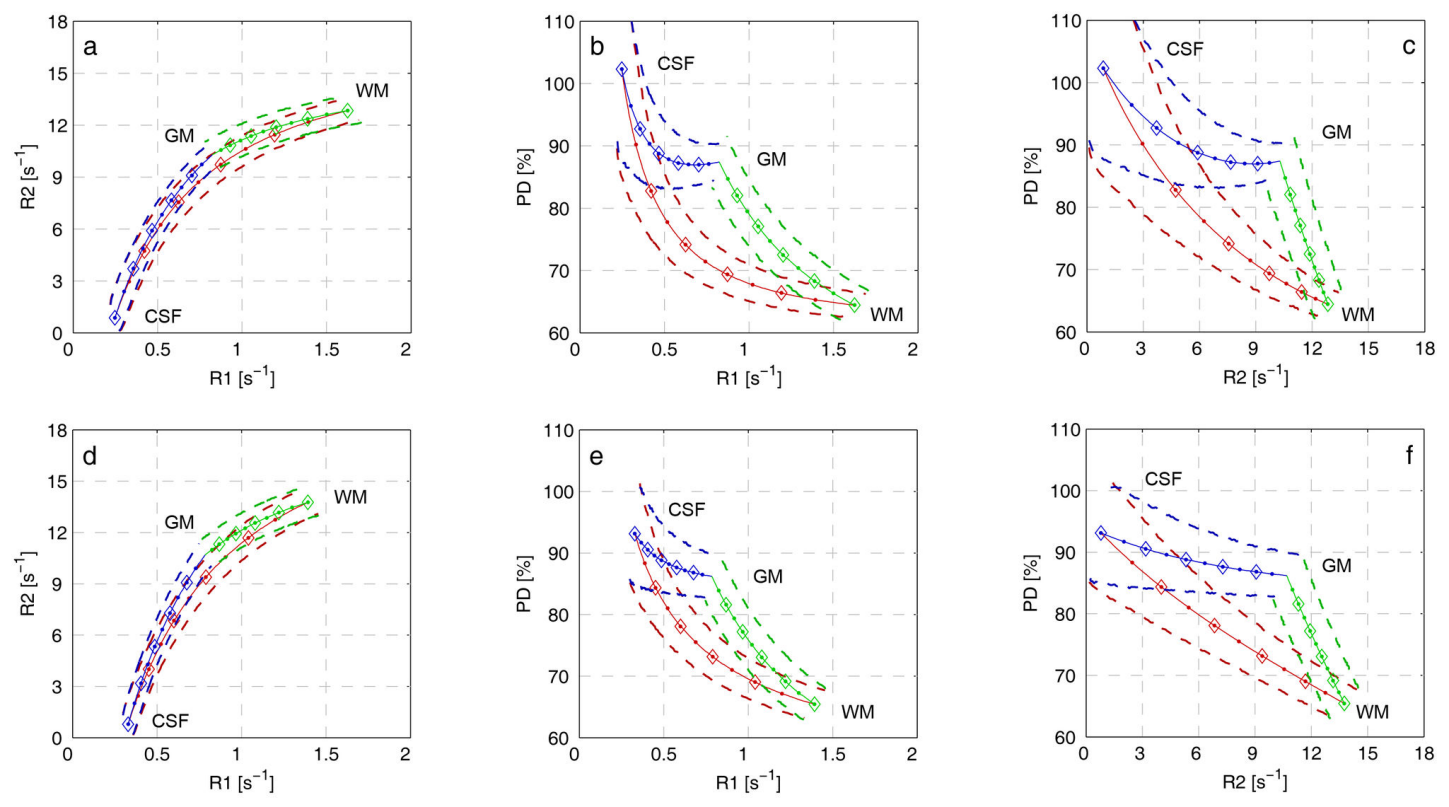
Lookup grids formed by joining together the 95% prediction ellipses of the 3,000 simulated partial-volume clusters (*dashed lines*). The transition GM↔WM is shown in green, WM↔CSF in red and WM↔CSF in blue. The top panels **a-c** shows the results for 1.5 T and the bottom panels **d-f** shows results for 3.0 T. The solid lines represent the observations in the absence of noise where each diamond corresponds to a change of 20% tissue fractions and each dot correspond to a change of 10% tissue fractions. R_1_-R_2_-PD space is projected on ***a*, *d*)** R_1_-R_2_, ***b*, *e*)** R_1_-PD and ***c*, *f*)** R_2_-PD planes.

The lookup grids, which were subsequently used in the brain tissue segmentation, were created from the measured pure tissue cluster positions in R_1_-R_2_-PD space and the partial volume tissue simulations. When the 95% prediction ellipses of the 3,000 simulated partial volume clusters were joined together in the R_1_-R_2_-PD space, curved bands were formed. In Figure 3 These lookup grids are shown for both field strengths. The significance of the bands is that 95% of brain voxels containing WM, GM and CSF, acquired with an SNR of 20, were expected to fall within the indicated region. Figure 3 also shows results from partial volume simulations without added noise. These results are shown as curves between the mean values of the pure tissue clusters.

**Figure 4:**
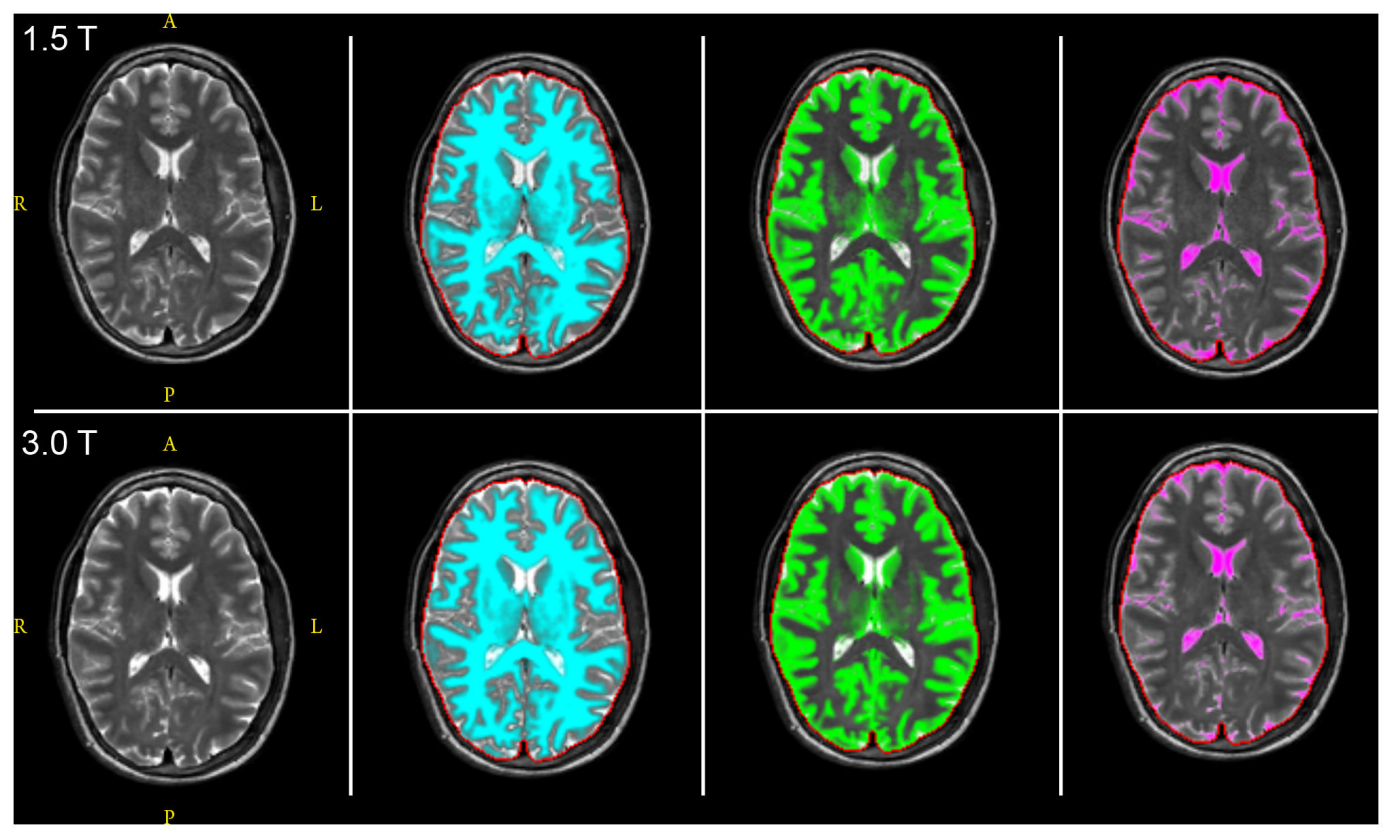
Brain tissue segmentation, of one slice in one healthy subject, at both 1.5 T (top panel) and 3.0 T (lower panel). From left to right: T2-weighted conventional image, white matter (blue), grey matter (green) and CSF (purple). The red lines are the brain intracranial mask calculated automatically by the SyMRI software.

**Figure 5:**
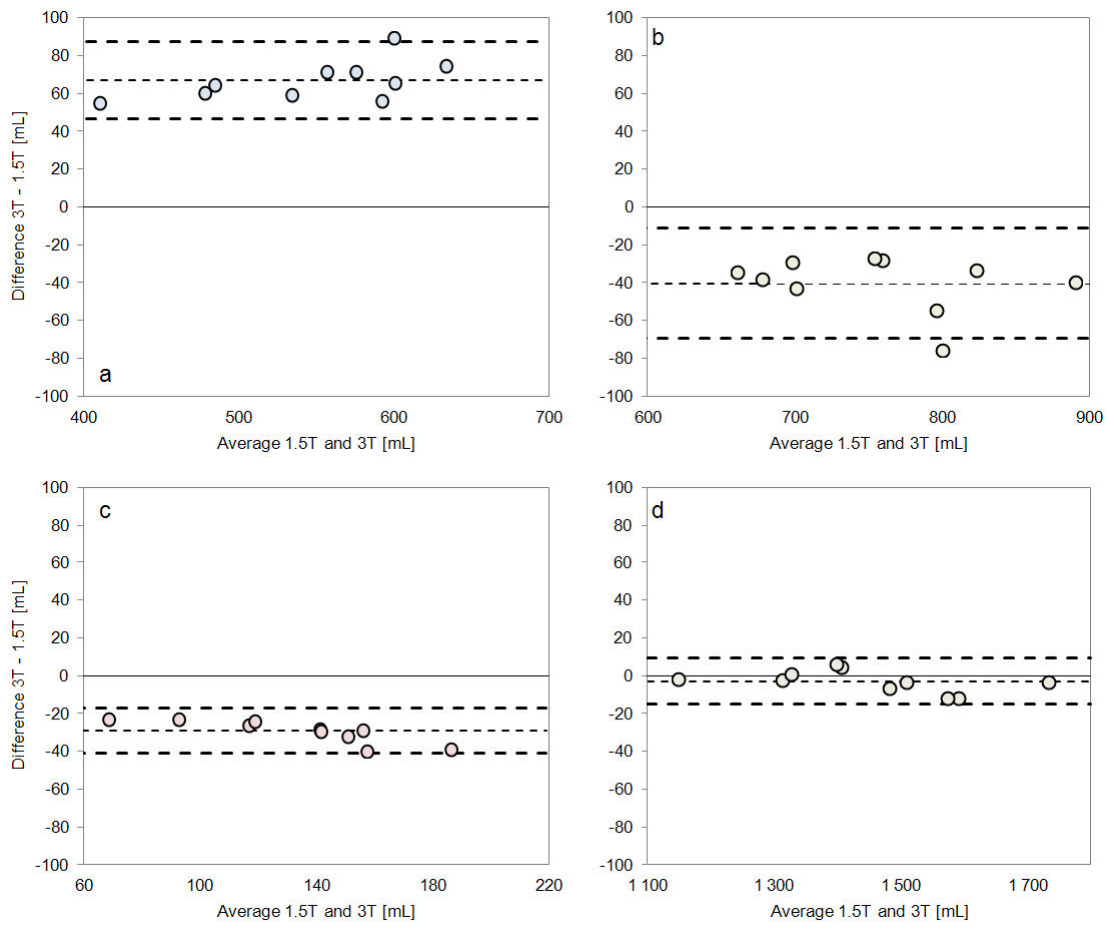
Bland-Altman plots comparing the volumes between 1.5 T and 3.0 T. Volumes of ***a*)** WM, ***b*)** GM, ***c*)** CSF and ***d*)** ICV for all 10 subjects measured at 1.5 T and 3.0 T. The dashed lines indicate the limits of agreement and the mean of all measurements (bias).

Representative results from WM, GM and CSF segmentation of the in vivo data acquired at both field strengths are shown in Figure 4. By visual inspection, the fully automated segmentation follows the contours of each tissue type as visualized in the T2-weighted images in the left panel. Table 2 lists segmentation results from the 1.5 T scanner, including the repeatability expressed in mL of tissue, and table 3 lists the corresponding segmentation results from the 3.0 T system. Within-subject standard deviation, repeatability and the coefficient of variation are also reported. Tissue volumes were normally distributed as assessed by the Shapiro-Wilk test. Figure 5 shows Bland-Altman plots comparing the 1.5 T and 3.0 T measurements for each subject and tissue type separately.

**Table 2:** Segmentation results for the 10 healthy subjects at the 1.5 T system.

**Subject (sex/age**)	**WM (mL**)	**GM (mL**)	**CSF (mL**)	**NON (mL**)	**ICV (mL**)	**BPV (mL**)	**BPF (%**)
F21	383 ± 0	679 ± 2	80 ± 0	9 ± 0	1151 ± 2	1071 ± 2	93.05 ± 0.07
M25	568 ± 0	840 ± 1	177 ± 0	12 ± 0	1598 ± 1	1420 ± 1	88.90 ± 0.00
M23	556 ± 3	839 ± 0	170 ± 0	15 ± 0	1580 ± 2	1410 ± 2	89.20 ± 0.00
M21	521 ± 7	824 ± 7	130 ± 1	11 ± 0	1486 ± 1	1357 ± 1	91.25 ± 0.07
F29	540 ± 3	697 ± 4	155 ± 1	13 ± 0	1405 ± 2	1250 ± 2	88.95 ± 0.07
F24	505 ± 3	774 ± 0	104 ± 0	13 ± 1	1396 ± 2	1292 ± 2	92.55 ± 0.07
M23	596 ± 1	911 ± 3	206 ± 1	22 ± 1	1735 ± 0	1530 ± 2	88.15 ± 0.07
F26	452 ± 8	723 ± 9	131 ± 2	8 ± 0	1314 ± 1	1183 ± 1	90.10 ± 0.14
F27	448 ± 3	713 ± 4	156 ± 0	10 ± 0	1328 ± 2	1171 ± 1	88.20 ± 0.00
F25	564 ± 1	768 ± 1	166 ± 3	12 ± 1	1511 ± 1	1345 ± 1	89.00 ± 0.14
sw	3.75	4.23	1.19	0.50	1.66	1.72	0.08
Repeatability	10.38	11.73	3.29	1.39	4.59	4.76	0.22
CV	0.0057	0.0043	0.0059	0.0329	0.0011	0.0013	0.0007

The columns list mean value and standard deviation from the first scan in the two sessions, for each subject. The last rows tabulate the within-subject standard deviation, *sw*, the repeatability and the coefficient of variation.

**Table 3:** Segmentation results for the 10 healthy subjects at the 3.0 T system.

**Subject (sex/age**)	**WM (mL**)	**GM (mL**)	**CSF (mL**)	**NON (mL**)	**ICV (mL**)	**BPV (mL**)	**BPF (%**)
F21	438 ± 6	644 ± 4	57 ± 1	9 ± 0	1149 ± 4	1092 ± 2	95.00 ± 0.14
M25	633 ± 9	807 ± 13	137 ± 0	8 ± 0	1586 ± 5	1448 ± 4	91.35 ± 0.07
M23	645 ± 25	763 ± 6	142 ± 6	18 ± 1	1568 ± 13	1426 ± 20	90.95 ± 0.49
M21	592 ± 4	769 ± 6	104 ± 1	14 ± 0	1480 ± 11	1375 ± 10	93.00 ± 0.00
F29	611 ± 5	659 ± 8	127 ± 9	12 ± 2	1409 ± 8	1282 ± 1	91.00 ± 0.57
F24	564 ± 7	746 ± 14	81 ± 2	10 ± 2	1402 ± 3	1321 ± 6	94.20 ± 0.14
M23	671 ± 1	871 ± 20	167 ± 15	23 ± 3	1732 ± 2	1565 ± 17	90.35 ± 0.92
F26	517 ± 17	680 ± 4	107 ± 0	8 ± 0	1312 ± 13	1205 ± 13	91.85 ± 0.07
F27	509 ± 4	684 ± 2	127 ± 9	9 ± 1	1329 ± 4	1202 ± 6	90.45 ± 0.64
F25	620 ± 3	741 ± 8	135 ± 4	11 ± 0	1508 ± 1	1373 ± 5	91.05 ± 0.21
Sw	10.59	9.95	6.78	1.34	7.83	10.26	0.44
Repeatability	29.33	27.57	18.77	3.71	21.70	28.42	1.21
CV	0.0141	0.0111	0.0371	0.0716	0.0045	0.0061	0.0036

The columns list mean value and standard deviation from the first scan in the two sessions, for each subject. The last rows tabulate the within-subject standard deviation, *sw*, the repeatability and the coefficient of variation.

GLM revealed statistically significant differences in total volume, between 1.5 T and 3.0 T, for WM (p < 0.001), GM (p < 0.001), CSF (p < 0.001), BPV (p < 0.001), and BPF (p < 0.001) but not for NON and ICV. Analyses of main effects showed that WM was underestimated, while GM and CSF were overestimated on 1.5 T compared to 3.0 T. The absolute differences and confidence intervals are listed in Table 4.

**Table 4:** Results from pair-wise comparisons of tissue volumes between the 1.5 T and the 3.0 T system.

**Dependent Variable**	**Mean Difference (mL**)** (1.5 T-3.0 T**)	**RSME**	**Mean Difference (% of 1.5 T**)**(1.5 T-3.0 T**)	**95% Confidence Interval of Difference (mL**)
WM	-66	68	-12.85%	[-72, -61]
GM	40	43	5.15%	[34,47]
CSF	29	30	19.66%	[26,33]
NON	0	2	0%	[-1, 1]
ICV	3	7	0.21%	[-1, 7]
BPV	-26	27	-2.00%	[-31, -22]
BPF (%)	-1.99	2.00	-2.21%	[-2.18, -1.79]

Relating the repeatability for each tissue type to the whole ICV (repeatability/ICV) is a relative measurement of repeatability with respect to the complete brain volume. Results for the 1.5 T system were 0.72% for WM, 0.81% for GM, 0.23% for CSF, 0.10% for NON, 0.32% for ICV and 0.33% for BPV. The corresponding numbers for 3.0 T were 2.03% for WM, 1.91% for GM, 1.30% for CSF, 0.26% for NON, 1.50% for ICV and 1.96% for BPV. The repeatability for BPF at 1.5 T was 0.22% and for 3.0 T 1.21%.

### Regional Analysis

**Figure 6:**
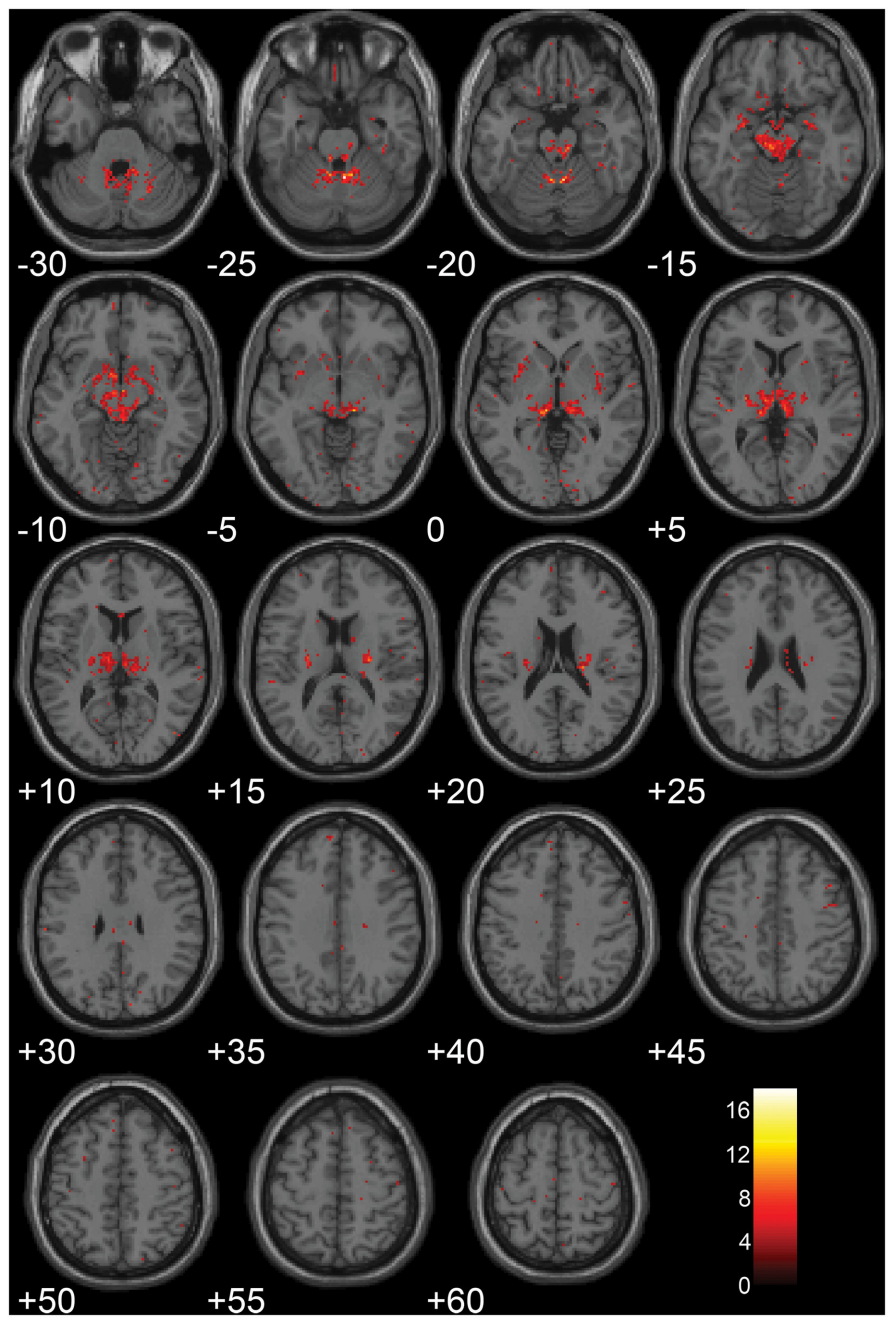
Results from voxel-wise t-tests comparing brain segmentation from all 10 subjects between 1.5 T and 3.0 T. Statistically significant differences are indicated where WM volume is estimated to be larger on the 3.0 T system.

The voxel-wise comparisons of tissue maps at 1.5 T and 3.0 T revealed regional differences in WM, GM and CSF volume estimations. In general, differences occurred in deep brain structures where tissue classified as mostly GM at 1.5 T was instead classified as mostly WM at 3.0 T. Statistically significant differences for CSF were only found in one small region close to the midline anterior cingulate gyrus in the limbic lobe (cluster size 26 voxels). Statistically significant regional differences in WM and GM estimation between 1.5 T and 3.0 T were found in bilateral and symmetrical regions in the following structures: the cerebellum, the anterior parts of the hippocampal formation, dorsal parts of the medulla and pons, midbrain structures comprising the thalamus, substantia nigra, red nucleus, and the dorsal midbrain tegmentum, as well as in several clusters in sub-lobar white and grey matter including the putamen. Figure 6 shows the results of the voxel-wise t-test where the WM estimate was significantly larger at 3.0 T compared to 1.5 T, the regions were GM estimates was significantly larger at 1.5 T was virtually identical.

## Discussion

In this study, one brain tissue segmentation and volume estimation method, previously reported for 1.5 T, was applied at both 1.5 T and 3.0 T Philips MR systems. It was shown that it was necessary to implement separate lookup grids (Figure 3) for the two field strengths since the tissue characteristics for WM, GM and CSF have varying R_1_-R_2_-PD values depending on main magnetic field strength (Table 1). Since the differences in tissue characteristics between the two field strengths were compensated for in the segmentation method, it was expected that tissue volumes would be similar at both field strengths, but variations were found. These may be caused by differences in regional tissue composition affecting the R_1_ and R_2_ values, differences in ROI placement in the field strengths or differences in the qMRI sequence implementation. These factors will be discussed in detail below.

First, regional tissue characteristics vary between 1.5 T and 3.0 T in a non-linear manner. This has been attributed to iron deposits in different anatomical regions [26,28], affecting mainly the measured R_2_, but also R_1_. Schenck et al. [28] showed that contrast between iron-rich and iron-depleted regions is substantially enhanced when the main magnetic field strength is increased (from 1.5 T to 4.0 T). This suggests that the differences in quantitative measurements between iron-rich and iron-depleted regions are also enhanced with field strength. Schenck et al. also found that the R_2_ dependence on field strength is greatest in regions with high iron concentration, and pointed out that the substantia nigra and the red nuclei are prominent indicators of this phenomenon. These structures were also found to differ using the present segmentation method. Schenck et al. showed a higher increase in T_1_ and T_2_ in the globus palladius, red nucleus, substantia nigra, putamen, thalamus and the splenium compared to the relatively iron-depleted regions of the midbrain. These discrepancies could possibly explain the misclassifications of the present algorithm in these particular regions. When determining tissue characteristics for the bulk WM, GM and CSF, as was done in the present study, local differences in R_1_ and R_2_ depending on anatomical structures were not included. This could lead to regional misclassifications when comparing field strengths. In this study the voxels which where differently classified in 1.5 T and 3.0 T closely resembled these reported anatomical regions. In a similar fashion, Pfefferbaum et al. [27] found regional volume differences between 1.5 T and 3.0 T, where some structures were measured as being up to 15% larger at 3.0 T, while other structures were instead down to 19% smaller, when using an SRI24 atlas-based approach. Pfefferbaum et al. thus concluded that regional volume differences were not due to simple (global) volumetric scaling. In another study Jovicich et al. [31] found, when using the FreeSurfer tool that both the magnitude and sign of regional volume differences depend on brain structure and field strength.

Second, since tissue characteristics vary between 1.5 T and 3.0 T, the appearance of conventional contrast-weighted MR images also varies. This may have led to differently interpreted boundaries between tissue types when ROIs were manually placed to obtain the prior knowledge of pure tissue clusters. The present method is to a degree sensitive to correctly measured pure tissue clusters and even small variations in ROI positioning on the two field strengths may have an effect on the total tissue volumes, calculated by the segmentation method. Identical ROI placement may be especially difficult to achieve in cortical GM, which is a relatively thin layer between WM and CSF. In one study, Han et al. [32] found that cortical thickness was detected to be 0.11 mm thicker in 3.0 T compared to 1.5 T in 15 subjects; something that suggests that GM ROIs may be easier to position at 3.0 T, compared to 1.5 T, and the pure tissue cluster for GM used in this study may differ between the two field strengths.

Third, even though the QRAPMASTER sequence is identically implemented at both field strengths, small differences may still exist due to such effects as a larger water-fat shift at 3.0 T, and slightly higher geometrical distortion due to the EPI readout. Moreover, B1-field inhomogeneities are larger on the 3.0 T MR scanner; thus residual inhomogeneities may have an effect.

The largest difference was observed in WM, while GM and CSF differed less between the two field strengths (Table 4). WM differences were observed up to 13%, GM up to 6% and CSF up to 20%. Since overestimating WM and underestimating GM have opposite effects on the BPV, the differences in BPV were notably smaller, only about 2%, although still statistically significant. The difference in ICV did not reach statistical significance between the field strengths. The BPF, which is the ratio of the BPV to the ICV, including CSF, showed a significantly higher value at 3.0 T. Even so, the results from this study indicated that the present segmentation method may reduce the differences in volume measurements between 1.5 T and 3.0 T system compared to FreeSurfer [31], SPM and Bayesian methods [33] for whole brain volumes.

In addition to the differences observed between the two scanners, we also investigated the repeatability in the two systems separately (Table 2 and Table 3). These results show that the repeatability is larger at 1.5 T for all measurements. This may be caused by the higher dynamic range in R_1_-R_2_ values on this system leading to greater separation of pure tissue clusters. Also, the MR head coil in the 3.0 T system was somewhat larger, allowing more movement by the subject, which could potentially lead to higher motion artefacts, affecting the repeatability.

### Limitations of This Study

This study was conducted on a relatively low number of healthy subjects in the age range of 20 to 30 years. The included subjects showed no signs of the degeneration or disease in the brain that would be expected in an elderly group or patient group. Such degeneration includes changes due to tissue atrophy, in particular around the ventricles, in addition to small, unspecific age-induced WM changes. It would therefore be interesting to extend the validation to other groups in future research.

This study was conducted using two different MR scanners. While effectively comparing these two scanners of different field strengths, the site-specific effect of the scanners themselves could not be separated in the statistical analysis. There is a possibility that some of the observed differences were not exclusively due to the field strength, but due to the individual scanners in question. Differences in scanners may include inhomogeneities, different gradient systems and differences in scanner design, system release and coils.

## Conclusions

The present study investigated the extension of a recently published brain tissue segmentation method, based on qMRI, to include both 1.5 T and 3.0 T field strengths. Validation was performed in 10 young and healthy subjects. The results showed that when compensating for global R_1_ and R_2_ differences, by implementation of separate lookup grids, most of the brain segmentation was identical at both field strengths and each scanner had high inner-scanner repeatability. However, regional differences in segmentation results were found in deep brain structures, the cerebellum, and the brain stem. These regional differences led to volume differences for each tissue type in the order of 40-70 mL (corresponding to about 5-20%) and differences in total brain volume of about 25 mL (2%). The total brain volume difference was less than that of the individual tissues, since differences in WM and GM had different sign. Therefore the total brain volume, where WM and GM were added together, was a more robust measure across field strengths. We hypothesize that some of the differences may have been caused by iron deposition, which previously has been shown to affect R_1_ and R_2_ values, in a non-linear manner. The present method is highly promising for application at both field strengths, but the local differences should preferably be accounted for before implementing the method in clinical practice or before combining field strengths in research studies. In future research it would be interesting to extend the present method using an atlas-based approach, with a locally adapting segmentation. Furthermore, as we have only examined relatively young adult healthy subjects, it would be of interest to also validate the segmentation method in selected groups with elderly subjects or in patients suffering from different neurological conditions.

## Supporting Information

Appendix S1 **Mathematical Details of Bloch Simulation.**
(DOCX)
