# Supplementary material for: Application of Quantitative MRI for Brain Tissue Segmentation at 1.5 T and 3.0 T Field Strengths
Source: PLoS One. 2013 Sep 16;8(9):e74795. doi: 10.1371/journal.pone.0074795 (PMC3774721; doi:10.1371/journal.pone.0074795)
Supplement: Appendix S1 — Mathematical Details of Bloch Simulation. (DOCX) [file pone.0074795.s001.docx]

**Appendix A: Mathematical Details of Bloch Simulation**

The numerical Bloch simulator simulated one single voxel, at a time, during the QRAPMASTER acquisition. The voxel contained 40 spin isochromats implemented as column vectors, each containing the magnetisation in the x, y and z direction, **M** = [*M_x_, M_y_, M_z_*]*^T^*. The spin isochromats were aligned at equidistant positions in the through-plane direction of a simulated 3-mm slice. The evolution of the spin magnetisation vectors were calculated in time steps of d*t* = 1 ns according to:

$\mathbf{M}_{n+1}=\mathbf{R}_{\mathrm{Re}lax}(t)*\mathbf{R}_{RF}(t)*\mathbf{R}_{GR}(d,t)*\mathbf{M}_{n}$ [2]

Where **R***_Relax_* described the relaxation behaviour of the spins, with the longitudinal relaxation rate R_1_ and the transverse relaxation rate R_2_, from the tissue being simulated, as:

$\left[\begin{matrix} M_{x} \\ M_{y} \\ M_{z} \end{matrix}\right]_{n+1}=\left[\begin{matrix} \exp(-R_{2}dt) & 0 & 0 \\ 0 & \exp(-R_{2}dt) & 0 \\ 0 & 0 & \exp(-R_{1}dt) \end{matrix}\right]*\left[\begin{matrix} M_{x} \\ M_{y} \\ M_{z} \end{matrix}\right]_{n}+\left[\begin{matrix} 0 \\ 0 \\ 1-\exp(-R_{1}dt) \end{matrix}\right]$ [3]

**R***_RF_* described the rotation around the x- or y-axis caused by the amplitude of the radio frequent RF pulse at each time point *t*. The rotation angle of **R***_RF_* was calculated as:

$\alpha(t)=A_{pulse}(t)*2\pi*\gamma*dt$ [4]

where A*_pulse_(t)* was the amplitude of the RF pulse at time point *t* and *γ* was the gyro-magnetic ratio (^1^H).

**R***_GR_* described the rotation around the z-axis caused by the gradient field strength, *S(t)* , at time point *t*. The rotation angle of **R***_GR_* was calculated as:

$\phi(d,t)=S(t)*d*2\pi*\gamma*dt$ [5]

Where *d* = 0 for the centre of the slice.

The initial value of the magnetisation for each spin isochromat at time t = 0 was set to
[*M_x_, M_y_, M_z_*]*^T^ = [0, 0, 1] ^T^*, representing thermal equilibrium.

The RF pulses, gradients and timings of the QRAPMSTER sequence were implemented as a script for the Bloch simulation. For each combination of saturation delay, *TD_i_*, and each echo time, *TE_j_*, the vector sum of all the spin isochromats was recorded, forming a matrix, **E**, with the magnetisation states, **M***_TDi,TEj_*:

$\mathbf{E}=\left[\begin{matrix} \mathbf{M}_{TD1,TE1} & \mathbf{M}_{TD1,TE2} & \ldots\\ \mathbf{M}_{TD2,TE1} & \mathbf{M}_{TD2,TE2} & \ldots\\ \vdots& \vdots& \ddots\end{matrix}\right]$ [6]

To simulate the presence of noise in the sequence, a matrix containing random white noise, **N**, in the interval [-1, 1] scaled with a factor 1/SNR, to reflect the SNR ratio of the measurement, was added to **E**:

$\mathbf{E}_{tissue}=\mathbf{E}+\mathbf{N}*1/SNR$ [7]

The magnetization state matrix, **E**, contained the simulated results of the complete QRAPMASTER acquisition, from the synthetically created tissue, in the same manner as raw data acquired directly from the MR scanner. All simulations were performed in MATLAB R2008a (MathWorks, 2008).
